# The Burden of Having to Wonder: Minority Stress Experiences of LGBTQ+ Hospice Family Caregivers

**DOI:** 10.1093/geroni/igab046.1379

**Published:** 2021-12-17

**Authors:** Kristin Cloyes, Miranda Jones, Marilisa Vega, Megan Thomas Hebdon, Casidee Thompson, Susan J Rosenkranz, Maija Reblin, Lee Ellington

**Affiliations:** 1 University of Utah, Portland, Oregon, United States; 2 University of Utah, Salt Lake City, Utah, United States; 3 University of Texas at Austin, University of Texas at Austin, Texas, United States; 4 Moffitt Cancer Center, Tampa, Florida, United States

## Abstract

Home hospice care relies heavily on informal caregivers, often patients’ family and close others. Hospice family caregivers report stress, burden, and unmet support needs associated with poor health and bereavement outcomes. These outcomes are sensitive to the quality of interactions with professional hospice providers, especially for historically marginalized groups, yet little research examines experiences of LGBTQ+ hospice family caregivers. Informed by minority stress theory, we conducted in-depth interviews with LGBTQ+ home hospice family caregivers across the U.S. (N=20). Participants reported demographics and described their caregiving experiences including interactions with hospice providers. Interviews were audio-recorded, transcribed, and content-analyzed. Participants were mostly white (n=15, 75%), non-Hispanic (n=19, 95%), cisgender (n=19, 95%), gender binary (n=19, 95%), lesbian (n=10, 50%), women (n=12, 60%); average age was 52.3 (range 25-67, SD=13.84). Along with known end-of-life caregiving stressors, participants experienced minority stress that complicated caregiver-provider communication. Distal stressors included lack of LGBTQ+ competent resources, inadequate legal protections, providers’ assumptions about relationships, and difficult dynamics with unaccepting relatives. Proximal stressors included perceived risks of disclosure, expectation of poor treatment, feeling the need to modify presentation of self or home, and wondering whether negative provider interactions were due to being LGBTQ+. This generated a background level of uncertainty, caution, and concern that was particularly distressing in the home setting. Minority stress affects LGBTQ+ people across the lifespan and generates added burdens and support needs for hospice family caregivers. Providers who understand these effects are better positioned to deliver safe, effective care to all families at end of life.

